# Supplementary material for: Flux Increase Occurring When an Ultrafiltration Membrane Is Flipped from a Normal to an Inverted Position—Experiments and Theory
Source: Membranes (Basel). 2022 Jan 21;12(2):129. doi: 10.3390/membranes12020129 (PMC8874773; doi:10.3390/membranes12020129)
Supplement: Supplementary file 1 [file membranes-12-00129-s001.zip › membranes-1546667-supplementary.pdf]

Supplementary Materials

Flux Increase that Occurs when Ultrafiltration Membrane is Flipped from Normal to Inverted Position - Experiments and Theory

Ladan Zoka [1], Ying Siew Khoo [2], Woei Jye Lau [2,*], Takeshi Matsuura [3,*], Roberto Narbaitz [1], Ahmad Fauzi Ismail [2]

[1] Department of Civil Engineering, University of Ottawa, 161 Louis Pasteur, Ottawa, ON, K1N 6N5, Canada
[2] Advanced Membrane Technology Research Centre (AMTEC), Universiti Teknologi Malaysia, 81310, Johor Bahru, Johor, Malaysia
[3] Department of Chemical and Biological Engineering, University of Ottawa, 161 Louis Pasteur, Ottawa, ON, K1N 6N5, Canada
* Correspondence: lwoeijye@utm.my (W.J. Lau); matsuura@uottawa.ca (T. Matsuura)

The derivation of Equation (17) in the main text is as follows.

Case 1: The top pore is large and the bottom pore is small. Subscript *t* and *b* in Equation (16) of the main text are replaced with *l* and *s*, respectively, and J is called $J_{l/s}$. Then,

$$J_{l/s} = \frac{\Delta p - \frac{k\rho v_l^2}{4} - \frac{\rho\alpha^2}{2} v_l^2 \left(\frac{r_l}{r_s}\right)^4}{\frac{8\eta\delta_s}{\pi\rho r_l^4}\left\{\left(\frac{\delta_l}{\delta_s}\right) + \left(\frac{r_l}{r_s}\right)^4\right\}} = \frac{\Delta p - \frac{\rho v_l^2}{4}\left\{k + 2\alpha^2\left(\frac{r_l}{r_s}\right)^4\right\}}{\frac{8\eta\delta_s}{\pi\rho r_l^4}\left\{\left(\frac{\delta_l}{\delta_s}\right) + \left(\frac{r_l}{r_s}\right)^4\right\}} \tag{S1}$$

Since

$$v_l = \frac{J_{l/s}}{\pi\rho r_l^2} \tag{S2}$$

$$J_{l/s} = \frac{\Delta p - \frac{\rho}{4}\left(\frac{J_{l/s}}{\pi\rho r_l^2}\right)^2 \left\{k + 2\alpha^2\left(\frac{r_l}{r_b}\right)^4\right\}}{\frac{8\eta\delta_s}{\pi\rho r_l^4}\left\{\left(\frac{\delta_l}{\delta_s}\right) + \left(\frac{r_l}{r_s}\right)^4\right\}} \tag{S3}$$

Both denominator and numerator is multiplied by r_l^4, then

$$J_{l/s} = \frac{\Delta p r_l^4 - \frac{\rho}{4}\left(\frac{J_{l/s}}{\pi\rho}\right)^2 \left\{k + 2\alpha^2\left(\frac{r_l}{r_b}\right)^4\right\}}{\frac{8\eta\delta_s}{\pi\rho}\left\{\left(\frac{\delta_l}{\delta_s}\right) + \left(\frac{r_l}{r_s}\right)^4\right\}} \tag{S4}$$

Now setting

$$P = \frac{\rho}{(\pi\rho)^2} \tag{S5}$$

$$Q = \frac{8\eta\delta_s}{\pi\rho} \tag{S6}$$

$$R = \left(\frac{r_l}{r_s}\right)^4 \tag{S7}$$

$$S = \frac{\delta_l}{\delta_s} \tag{S8}$$

$$T1 = k + 2\alpha^2\left(\frac{r_l}{r_b}\right)^4 \tag{S9}$$

$$J_{l/s} = \frac{\Delta p {r_l}^4 - \frac{PT1}{4}\left(J_{l/s}\right)^2}{Q(S+R)} \tag{S10}$$

Hence,

$$\frac{PT1}{4}\left(J_{l/s}\right)^2 + Q(S+R)J_{l/s} - \Delta p {r_l}^4 = 0 \tag{S11}$$

Solving the quadratic equation for $J_{l/s}$,

$$J_{l/s} = \frac{-Q(S+R)+\sqrt{Q^2(S+R)^2+PT1\Delta p {r_l}^4}}{\frac{PT1}{2}} \tag{S12}$$

Case 2: The membrane is flipped. Now the top pore is small and the bottom pore is large.

From Equation (16) of the main text

$$J_{s/l} = \frac{\Delta p - \frac{k\rho v_s^2}{4} - \frac{\rho\alpha^2}{2}v_s^2\left(\frac{r_s}{r_l}\right)^4}{\frac{8\eta\delta_l}{\pi\rho {r_s}^4}\left\{\left(\frac{\delta_s}{\delta_l}\right)+\left(\frac{r_s}{r_l}\right)^4\right\}} = \frac{\Delta p - \frac{\rho v_l^2}{4}\left\{k\left(\frac{r_l}{r_s}\right)^4+2\alpha^2\right\}}{\frac{8\eta\delta_l}{\pi\rho {r_s}^4}\left\{\left(\frac{\delta_s}{\delta_l}\right)+\left(\frac{r_s}{r_l}\right)^4\right\}} \tag{S13}$$

$$J_{s/l} = \frac{\Delta p - \frac{\rho v_l^2}{4}\left\{k\left(\frac{r_l}{r_s}\right)^4+2\alpha^2\right\}}{\frac{8\eta\delta_l}{\pi\rho {r_s}^4}\left\{\left(\frac{\delta_s}{\delta_l}\right)+\left(\frac{r_s}{r_l}\right)^4\right\}} = \frac{\Delta p - \frac{\rho}{4}\left(\frac{J_{s/l}}{\pi\rho {r_l}^2}\right)^2\left\{k\left(\frac{r_l}{r_s}\right)^4+2\alpha^2\right\}}{\frac{8\eta\delta_l}{\pi\rho {r_s}^4}\left\{\left(\frac{\delta_s}{\delta_l}\right)+\left(\frac{r_s}{r_l}\right)^4\right\}} \tag{S14}$$

Multiplying both denominator and numerator with ${r_l}^4$,

$$J_{s/l} = \frac{\Delta p{r_l}^4 - \frac{\rho}{4}\left(\frac{J_{s/l}}{\pi\rho}\right)^2\left\{k\left(\frac{r_l}{r_s}\right)^4 + 2\alpha^2\right\}}{\frac{8\eta\delta_l {r_l}^4}{\pi\rho {r_s}^4}\left\{\frac{\delta_s}{\delta_l} + \left(\frac{r_s}{r_l}\right)^4\right\}} = \frac{\Delta p{r_l}^4 - \frac{\rho}{4}\left(\frac{J_{s/l}}{\pi\rho}\right)^2\left\{k\left(\frac{r_l}{r_s}\right)^4 + 2\alpha^2\right\}}{\frac{8\eta\delta_s}{\pi\rho}\left\{\left(\frac{\delta_l}{\delta_s}\right) + \left(\frac{r_l}{r_s}\right)^4\right\}} \quad \text{(S15)}$$

Using Equations (S5) to (S8)

and setting

$$T2 = k\left(\frac{r_l}{r_s}\right)^4 + 2\alpha^2 \quad \text{(S16)}$$

$$J_{s/l} = \frac{\Delta p{r_l}^4 - \frac{PT2}{4}\left(J_{s/l}\right)^2}{Q(S+R)} \quad \text{(S17)}$$

Rearranging,

$$\frac{PT2}{4}\left(J_{s/l}\right)^2 + Q(S+R)J_{s/l} - \Delta p{r_l}^4 = 0 \quad \text{(S18)}$$

Solving the quadratic equation,

$$J_{s/l} = \frac{-Q(S+R) + \sqrt{Q^2(S+R)^2 + PT2\Delta p{r_l}^4}}{PT2/2} \quad \text{(S19)}$$

From Equations (S12) and (S19),

$$\frac{J_{l/s}}{J_{s/l}} = \frac{\left(\frac{-Q(S+R) + \sqrt{Q^2(S+R)^2 + PT1\Delta p{r_l}^4}}{PT1/2}\right)}{\left(\frac{-Q(S+R) + \sqrt{Q^2(S+R)^2 + PT2\Delta p{r_l}^4}}{PT2/2}\right)} = \frac{T2}{T1} \times \frac{-Q(S+R) + \sqrt{Q^2(S+R)^2 + PT1\Delta p{r_l}^4}}{-Q(S+R) + \sqrt{Q^2(S+R)^2 + PT2\Delta p{r_l}^4}} \quad \text{(S20)}$$

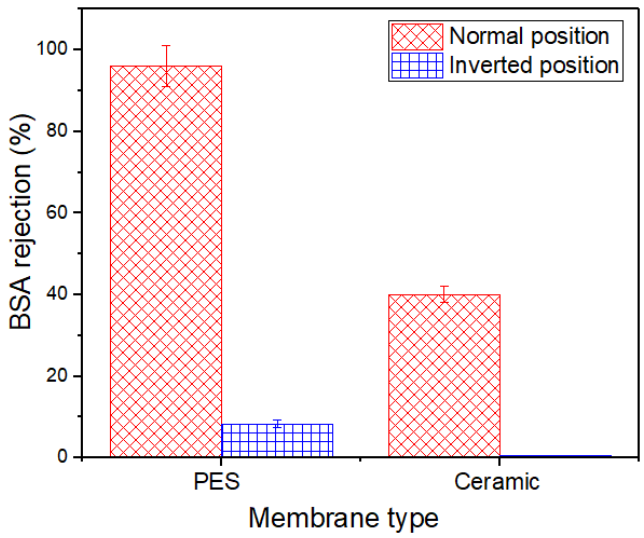

Figure S1. Comparison between the BSA rejection of PES and ceramic membrane in normal and inverted position.

Table S1. BSA concentration in feed and permeate samples produced from PES and ceramic membrane in normal and inverted position.

Membrane	Position	BSA Feed (ppm)	BSA Permeate (ppm)
PES	Normal	100	4.0
	Inverted	100	91.7
Ceramic	Normal	500	300
	Inverted	500	490
